# Complete Genome Sequence of a Novel Myovirus Which Infects Atypical Strains of *Edwardsiella tarda*

**DOI:** 10.1128/genomeA.00248-12

**Published:** 2013-02-28

**Authors:** Motoshige Yasuike, Emi Sugaya, Yoji Nakamura, Yuya Shigenobu, Yasuhiko Kawato, Wataru Kai, Satoshi Nagai, Atushi Fujiwara, Motohiko Sano, Takanori Kobayashi, Toshihiro Nakai

**Affiliations:** a Research Center for Aquatic Genomics, National Research Institute of Fisheries Science, Fisheries Research Agency, Yokohama, Japan; b Department of Marine Biotechnology, Faculty of Life Science and Biotechnology, Fukuyama University, Fukuyama, Hiroshima, Japan; c Research Center for Fisheries Oceanography and Marine Ecosystem, National Research Institute of Fisheries Science, Fisheries Research Agency, Yokohama, Japan; d Graduate School of Biosphere Science, Hiroshima University, Higashihiroshima, Japan

## Abstract

We present the genome sequence of a novel *Edwardsiella tarda*-lytic bacteriophage, MSW-3, which specifically infects atypical *E. tarda* strains. The morphological and genomic features of MSW-3 suggest that this phage is a new member of the dwarf myoviruses, which have been much less studied than other groups of myoviruses.

## GENOME ANNOUNCEMENT

*Edwardsiella tarda* is a Gram-negative bacterium and the causative agent of edwardsiellosis, which affects a wide range of hosts including humans and a variety of animals. Particularly, edwardsiellosis has caused severe economic losses in both marine and freshwater fish farms worldwide (reviewed in reference 1). Two motile phenotypic strains of *E. tarda*, which also differ in carbohydrate utilization and pathogenicity, have been isolated from various kinds of diseased fishes. Typical (motile) strains have been isolated mainly from freshwater fish and Japanese flounder (*Paralichthys olivaceus*), while atypical (non-motile) strains have been isolated mainly from red sea bream (*Pagrus major*) and yellowtail (*Seriola quinqueradiata*) (2–4).

Bacteriophages (phages) infecting typical and/or atypical strains of *E. tarda* have been isolated from various fish tissues and seawater samples from fish farms (5). Recently, we have reported the complete genome sequence of two *E. tarda*-lytic podoviral phage isolates that infected a wide range of *E. tarda* strains, including both the typical and atypical strains (6). To further understand phage-host interaction mechanisms, we determined the complete genome sequence of a novel *E. tarda*-lytic phage, MSW-3, which specifically infected atypical *E. tarda* strains.

We isolated MSW-3 from a seawater sample obtained from a red sea bream farm. Whole-genome shotgun sequencing of MSW-3 was performed using Roche 454 GS-FLX titanium pyrosequencing. *De novo* assembly of sequence reads was performed using a 454 Newbler 2.5.3, and open reading frames (ORFs) were predicted using GeneMarkS (7) and Glimmer3 (8). The predicted ORFs were annotated using BLASTP (9) against the viral sequence database (E value threshold of 1E^–3^).

Electron microscopic observations of MSW-3 showed a myovirus morphology (the family *Myoviridae*) with a head length of 60 nm and a contractile tail length of 80 nm (10). This phage size is smaller than that of the typical myoviruses. For example, the well-studied myovirus phage T4 has a head of 111 nm and a tail of 113 nm (11). The size of the entire MSW-3 genome was 42,746 bp. This genome size is also smaller than that of most other myovirus genomes, whose genome sizes are over 160 kb (12). Sixty-six ORFs were predicted in the MSW-3 genome and twenty-two ORFs (31.9%) share homology with protein sequences from iodobacteriophage ϕPLPE (24.4 to 68.2% identities and 42.9 to 81.9% similarities). ϕPLPE infects a Gram-negative bacterium of the genus *Iodobacter* that commonly inhabits aquatic environments such as rivers, streams, and canals (13). ϕPLPE also is a small myovirus, with a genome size of 47,453 bp (13). Recently, small myoviruses like ϕPLPE have been described as “dwarf myoviruses,” those with a genome size of less than 50 kb (14). Thus, these morphological and genomic features of MSW-3 suggest that MSW-3 is a new member of dwarf myoviruses. The complete genome information of MSW-3 and the two existing *E. tarda* podoviral phages (6) will advance our understanding of the molecular mechanisms involved in their host specificity.

### Nucleotide sequence accession number. 

The complete genome sequence of the *E. tarda* phage MSW-3 was submitted to DDBJ under the accession number AB767244.
